# Placental Pathology Findings in Unexplained Pregnancy Losses

**DOI:** 10.1007/s43032-023-01344-3

**Published:** 2023-09-19

**Authors:** Beatrix B. Thompson, Parker H. Holzer, Harvey J. Kliman

**Affiliations:** 1 Department of Obstetrics, Gynecology and Reproductive Sciences, Yale University School of Medicine, New Haven, CT USA; 2 Harvard Medical School, Boston, MA USA; 3 Department of Statistics & Data Science, Yale University, New Haven, CT USA; 4 Spiff Incorporated, Sandy, UT USA

**Keywords:** Pregnancy loss, Miscarriage, Stillbirth, Placenta, Trophoblast inclusion, Small placenta

## Abstract

There are approximately 5 million pregnancies per year in the USA, with 1 million ending in miscarriage (a loss occurring prior to 20 weeks of gestation) and over 20,000 ending in stillbirth at or beyond 20 weeks of gestation. As many as 50% of these losses are unexplained. Our objective was to evaluate the effect of expanding the placental pathology diagnostic categories to include the explicit categories of (1) dysmorphic chorionic villi and (2) small placenta in examining previously unexplained losses. Using a clinical database of 1256 previously unexplained losses at 6–43 weeks of gestation, the most prevalent abnormality associated with each loss was determined through examination of its placental pathology slides. Of 1256 cases analyzed from 922 patients, there were 878 (69.9%) miscarriages and 378 (30.1%) antepartum stillbirths. We determined the pathologic diagnoses for 1150/1256 (91.6%) of the entire series, 777/878 (88.5%) of the miscarriages (< 20 weeks’ gestation), and 373/378 (98.7%) of the stillbirths (≥ 20 weeks’ gestation). The most common pathologic feature observed in unexplained miscarriages was dysmorphic chorionic villi (757 cases; 86.2%), a marker associated with genetic abnormalities. The most common pathologic feature observed in unexplained stillbirths was a small placenta (128 cases; 33.9%). Our classification system reinforced the utility of placental examination for elucidating potential mechanisms behind pregnancy loss. The improved rate of diagnosis appeared to be the result of filling a gap in previous pregnancy loss classification systems via inclusion of the categories of dysmorphic chorionic villi and small placenta.

**Graphical Abstract:**

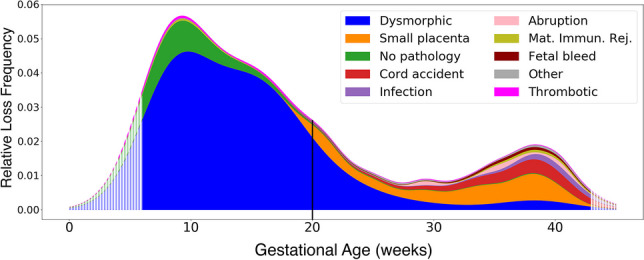

## Introduction

Miscarriage rates based on life table analysis reveal that the cumulative risk of pregnancy loss between 5 and 20 weeks of gestation ranges between 11 and 22% [1]. Although pregnancy loss rates decrease after 20 weeks of gestation, there are approximately 2 million stillbirths globally per year [2], with over 20,000 losses occurring annually in the USA [3, 4]. Up to 60–70% of miscarriages are caused by aneuploidies [5–7], and although many of these cases were historically classified as unexplained, recent detailed studies have steadily increased the genetic fraction [8–10]. Despite these advances, the pressing clinical issue remains identifying the cause of the loss and employing methods of preventing future losses when possible [11–14].

Current pregnancy loss classification systems require improved consistency to more accurately determine the potential causes of each pregnancy loss [11, 15]. A 2009 systemic review found a large variability in the rates of unexplained stillbirths when various classification systems were applied to the same cohort of stillbirths, ranging from 9.5 to 50.4% [15]. Consistent with these findings, the Centers for Disease Control and Prevention’s 2015–2017 Cause of Fetal Death report found that the most frequent cause for fetal death was “Unspecified.” [16].

The key may be the placenta, as placental abnormalities are commonly detected in adverse pregnancy outcomes [11, 17–20], and have been associated with potentially preventable types of losses [21–23]. One systemic review reported that up to 65% of stillbirths are attributable to placental abnormalities [24]. However, absent in these abovementioned classification systems are the categories of dysmorphic chorionic villi, represented by trophoblast inclusions [20, 25–37], and the consistent inclusion of the category of a small placenta, which is clearly associated with pregnancy loss [38–41]. We thus hypothesized that expanding the placental pathology diagnostic categories to include the two explicit categories of dysmorphic chorionic villi and small placenta in examining previously unexplained losses could decrease the number of cases that remained “Unspecified” [16].

## Materials and Methods

### Cases

A case series of 1527 singleton pregnancies that ended in loss were identified from our tertiary-care consult service. Cases were excluded if the cause of loss could be elucidated from the clinical records alone, such as the presence of aneuploidies. Available demographic, clinical data, and gross description were abstracted from the clinical records when submitted with the consult request. Hematoxylin and eosin placental slides (no autopsy slides) were reviewed by the senior author (HJK). The analysis of this retrospective case series was approved by the Yale University Human Research Protection Program Institutional Review Board (protocol ID 2000029781).

### Excluded Cases

**Fig. 1:**
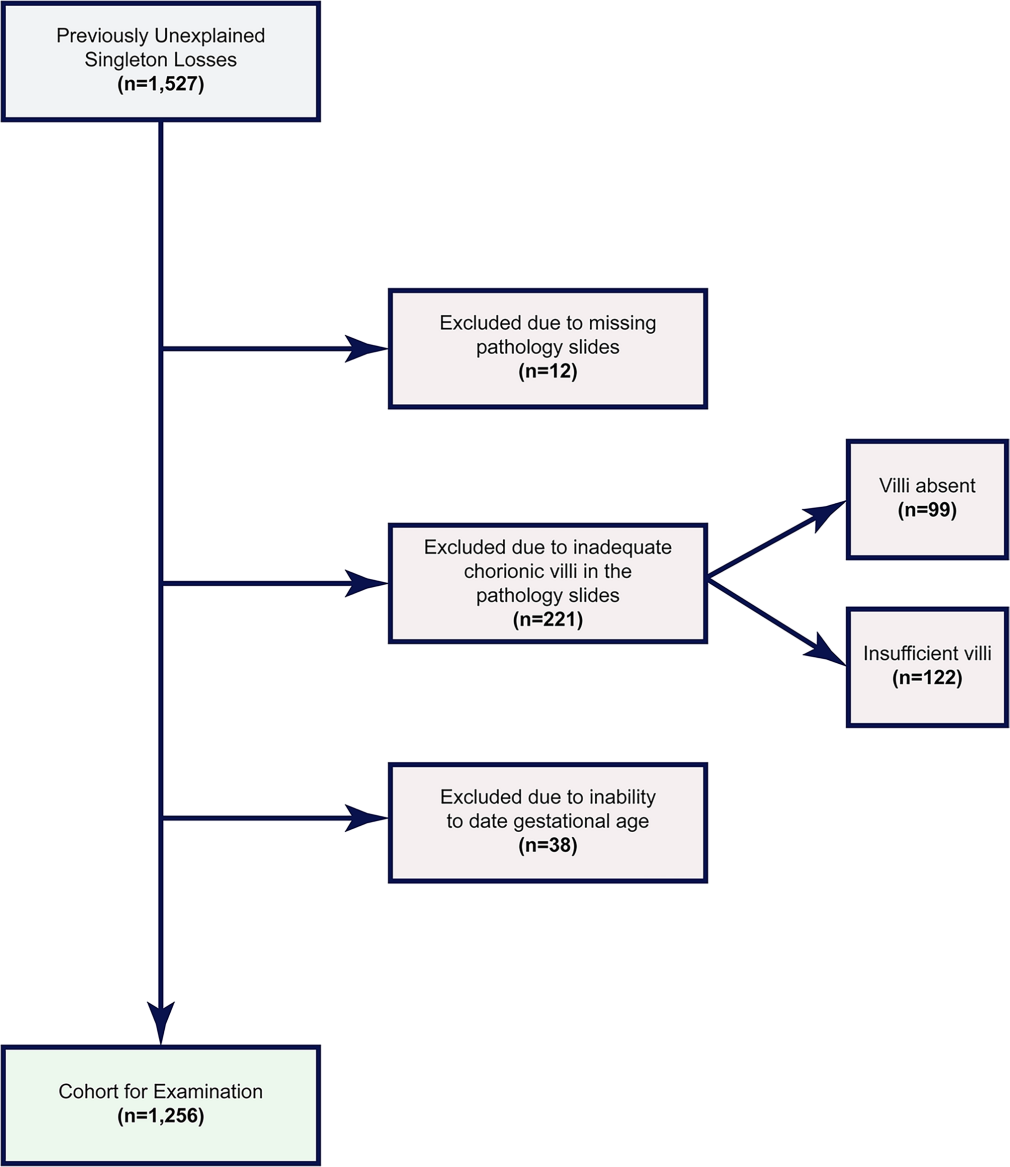
Flowchart demonstrating the selection and exclusion criteria of the eligible cases

Cases with missing pathology slides, or an absence or insufficient number (fewer than five cross sections) of chorionic villi in the placental sample (Fig. 1) were excluded. The second exclusion criterion was an inability to date the clinical gestational age (GA), determined by the patient’s last menstrual period (LMP). In the absence of an LMP, the GA was approximated by chorionic villus histologic criteria [42–44]. The remaining cases in which gestational age could not be reliably estimated were excluded from further analysis. All subsequent references to GA are related to LMP dating.

### Pathologic Evaluation

**Fig. 2:**
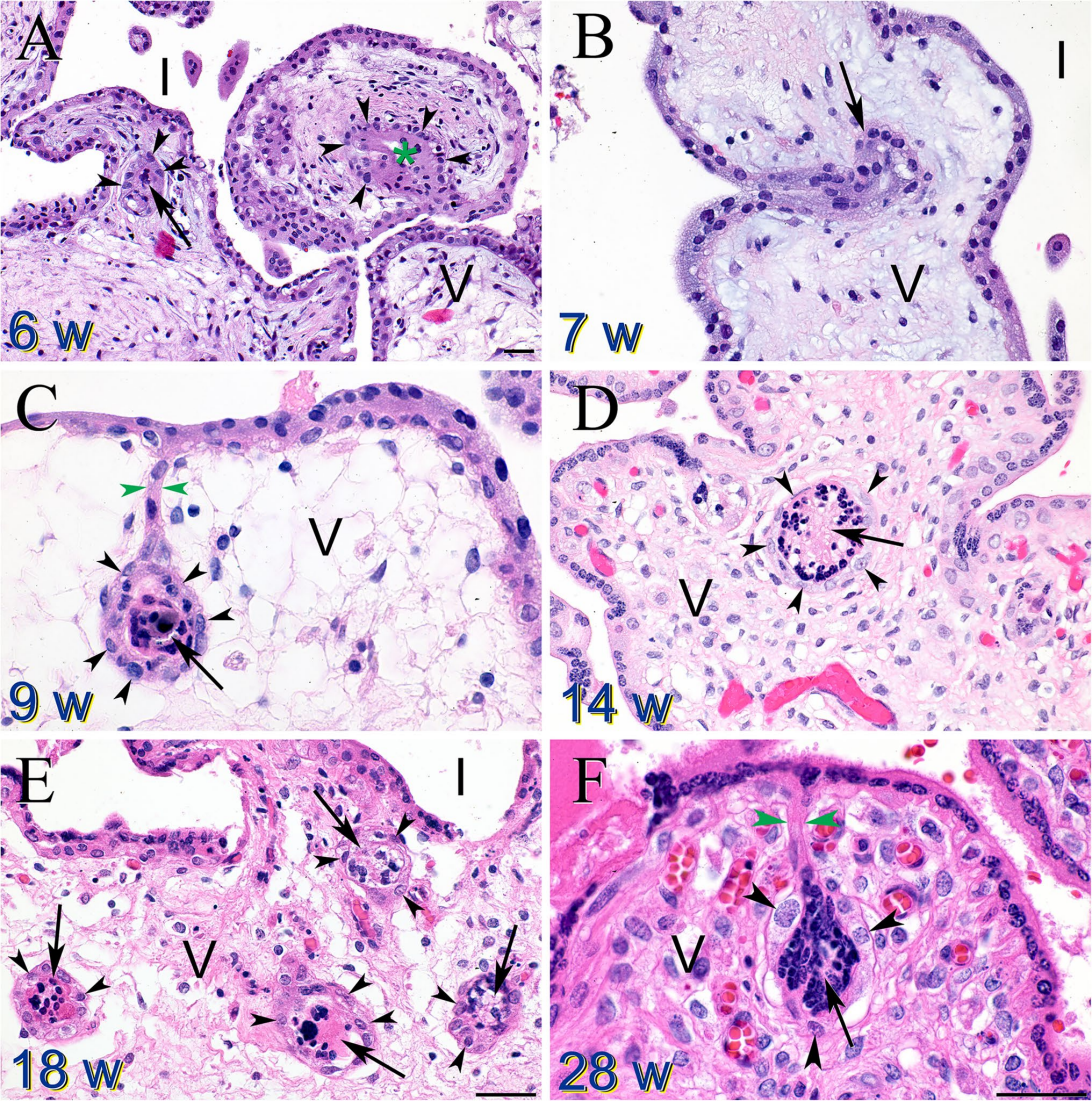
Trophoblast invaginations and inclusions (TIs). **A** Chorionic villi from a 6-week loss revealed both a trophoblast inclusion (TI) (arrow) and invagination (green asterisk), with surrounding cytotrophoblasts (black arrowheads). Intervillous space (I) and villus core (V). **B** Trophoblast invagination (arrow) in a villus from a 7-week loss. **C** Trophoblast invagination (green arrowheads) forming a TI (arrow) with surrounding cytotrophoblasts (black arrowheads). **D** Cross section of a large TI (arrow) surrounded by cytotrophoblasts (black arrowheads) in a 14-week villus. **E** Multiple TIs (arrows) with surrounding cytotrophoblasts (black arrowheads) in an 18-week villus. **F** Trophoblast invagination (green arrowheads) forming a TI (arrow) with surrounding cytotrophoblasts (black arrowheads). Magnification bars all represent 50 μM. Panels B–E are all at the same magnification

The placental pathology of included cases was re-reviewed following the Amsterdam Placental Workshop Group Consensus Statement [45], with the following modifications. This statement does not include the diagnostic categories of dysmorphic chorionic villi, trophoblast inclusions (TIs), and/or invaginations (Fig. 2). TIs were first described by Boyd and Hamilton in 1964 [46], and later linked specifically to placentas from triploid losses in 1969 [47, 48]. Over time other investigators found that TIs were not a specific marker of triploidy but rather were seen in a wide range of karyotypic and non-karyotypic genetic abnormalities [25, 27–30, 49, 50], and adverse pregnancy outcomes, including stillbirth [20]. Importantly, the frequency of TIs in normal control placentas is very low [51–53]. Therefore, we added dysmorphic chorionic villi (not to be confused with villous dysmaturity [45]) as a diagnostic category, defined as identification of at least one TI and/or multiple invaginations in the examined slides. Additionally, based on normative curves developed by Pinar et al. [54], we added the explicit category of small placenta, defined as fixed trimmed disk weight below the 10th percentile for cases ≥ 20 weeks. Values below the 10th percentile were mathematically extrapolated from the primary Pinar data.

**Fig. 3:**
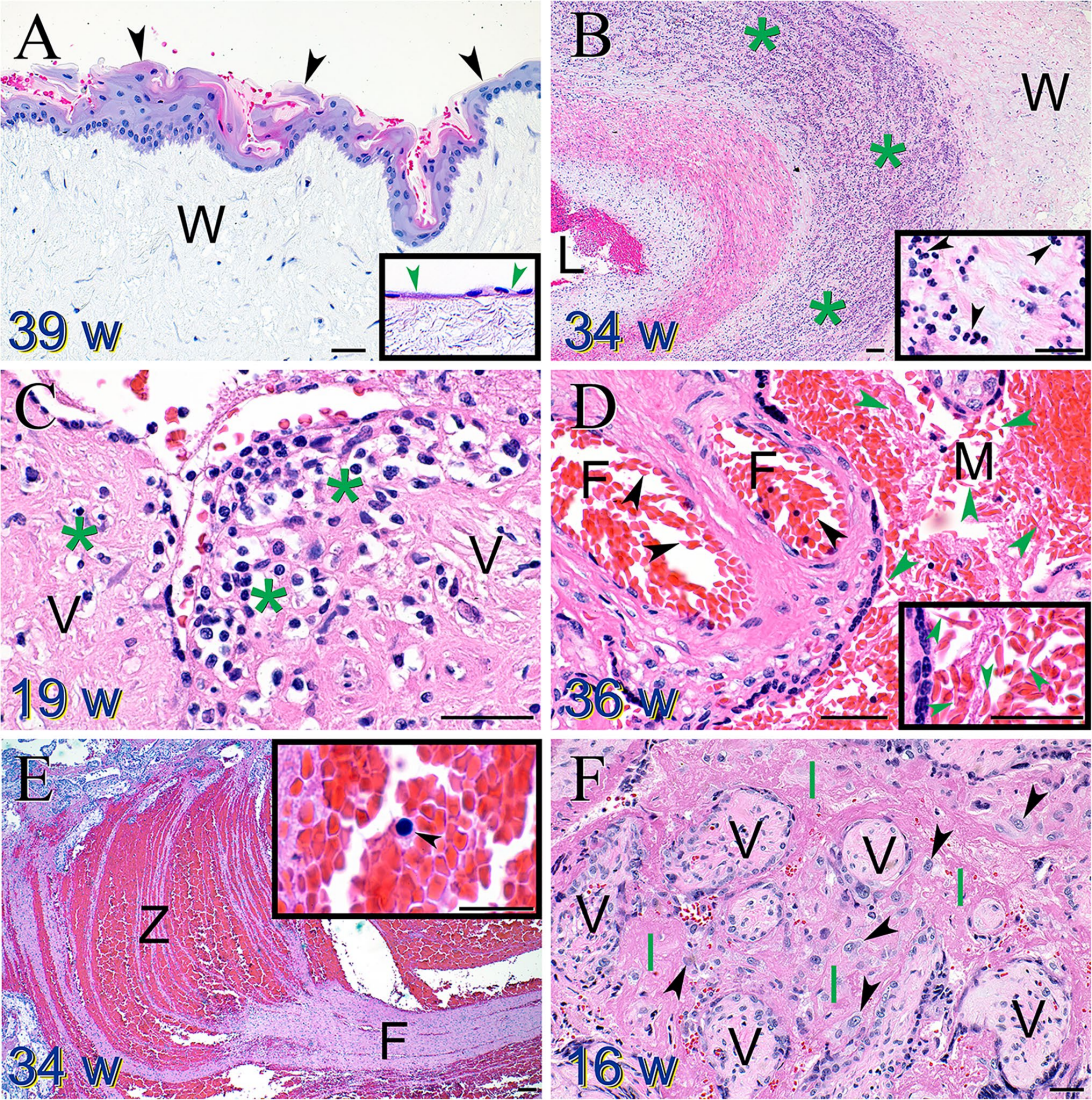
Select placental pathology findings in pregnancy losses. **A** Squamous metaplasia of the umbilical cord surface epithelium (black arrowheads) confirms compression in this 39-week gestation. Compare to the normal umbilical cord surface epithelium (green arrowheads) in the inset. Wharton’s jelly (W). Magnification bar = 50 μM. **B** Severe fetal inflammatory response observed as a large wave of neutrophils (green asterisks) migrating through and into the Wharton’s jelly (W) of a 34-week gestation umbilical vein. Magnification bar = 100 μM. Inset reveals individual neutrophils (black arrowheads). Magnification bar = 50 μM. **C** Maternal T-cells (green asterisks) infiltrating into a 19-week chorionic villus (V). Magnification bar = 50 μM. **D** Sickled erythrocytes observed in both the fetal (F) (black arrowheads) and maternal (M) circulations (green arrowheads) in a 36-week gestation. Magnification bars = 50 μM. **E** Fetal maternal hemorrhage evidenced by a large area of intervillous thrombosis with characteristic regions of fibrin (F) and lines of Zahn (Z). Magnification bar = 100 μM. Inset reveals a nucleated fetal erythrocyte (black arrowhead). Magnification bar = 25 μM. **F** Villi (V) from a 16-week gestation are totally enmeshed in intervillous fibrin (referred to as massive perivillous fibrin)—a result of maternal intervillous blood thrombosis (I). Detached villus trophoblasts (black arrowheads) can be seen migrating through the fibrin. Magnification bar = 50 μM

Identifying a nonacute cord accident required evidence of cord compression, as manifested by (1) the presence of squamous metaplasia [55–57] on the umbilical cord surface (Fig. 3A); (2) fetal hypoxia defined as an abnormal increase in fetal nucleated RBCs [58]; (3) and thrombosis within the fetal circulation [59]. A loss was only identified as being caused by an infection when a fetal inflammatory response was observed, evidenced by either fetal neutrophil migration through the fetal chorionic plate vessels and/or through the umbilical cord vessels (funisitis) (Fig. 3B) [60]. A maternal inflammatory response alone, as evidenced by maternal neutrophils migrating into and through either the chorionic plate or external membranes, was not sufficient to identify a loss as being caused by an infection. Maternal immunologic rejection was identified when significant numbers of maternal T-cells infiltrated the chorionic villi (chronic villitis, Fig. 3C) [61–64], or monocytes filled the intervillous space (chronic histiocytic intervillositis; CHI) [65–67]. Abruption occurred when a clear, well-developed fibrin clot was adherent to the maternal surface of the placenta [68]. Fetal maternal hemorrhage was identified when intervillous fibrin forming layered lines of Zahn (indicative of blood clot formation in flowing blood [69]) was admixed with blood containing nucleated red blood cells (indicative of a fetal bleeding source) (Fig. 3E) [70, 71]. In contrast, massive perivillous fibrin (a manifestation of maternal intervillous blood thrombosis [72–74]) was identified when the intervillous space was largely filled with fibrin (Fig. 3F).

### Classification System

After pathologic examination, we identified the most prevalent abnormality associated with the loss according to the following classification system. First, any clear and marked case of abruption, cord accident, or fetal bleed was assigned. Next, we identified all cases with evidence of thrombosis or fetal inflammatory response.

After losses associated with the above five abnormalities were identified, the remaining cases with a placental weight < 10th percentile for the corresponding gestational age were categorized as a small placenta and sorted into four etiologic sub-categories: small placenta with evidence of maternal immunologic rejection, small placenta with dysmorphic chorionic villi, small placenta with evidence of uteroplacental insufficiency (evidenced by findings of increased syncytial knots and accelerated maturation of the chorionic villi), or small placenta with no other pathologic findings.

Next, remaining cases with indication of maternal immunologic rejection were classified. Cases that showed dysmorphic chorionic villi with no other etiology were then assigned. The remaining “other” defined abnormalities included viral stigmata revealed on pathologic examination [75, 76], uteroplacental insufficiency without a concomitantly small placenta [77], maternal and/or fetal sickle cell disease (Fig. 3D) [78], premature inappropriate maternal perfusion prior to 8 weeks of gestation [79], complete mole [27], and severe intraamniotic fluid infection without apparent fetal inflammatory responses [80]. Cases revealing no pathologic findings remained unexplained.

### Statistical Analysis

We displayed the distribution of pregnancy losses across gestational age and associated abnormalities using kernel density estimation [81, 82]. This smoothed version of a histogram that replaces each individual data point is replaced with a Gaussian and the total density plot is the sum of all such Gaussians. For each individual category, all corresponding gestational ages were used to create a density estimate of that associated abnormality. Then, to account for how some abnormalities occur more frequently than others, we multiplied the density of each cause by the proportion of cases with that associated abnormality.

To analyze the frequencies of small and large placentas in our series, we converted placental weight percentiles to *z*-scores, allowing us to visualize this loss cohort against the standard *z*-score distribution of placentas from normal term or uncomplicated preterm deliveries [54].

We conducted an analysis of patients with multiple losses to investigate whether their associated abnormalities were correlated. More precisely, the null hypothesis to be tested was that the abnormality identified in the second loss was not related to that of the first loss. We tested this against the alternative hypothesis that the abnormality identified in the second loss was the same as that of the first. To perform this hypothesis test, we used a permutation test [83]. Specifically, we randomly shuffled the order of all second losses and calculated what proportion of them matched the findings in the unshuffled first loss causes. Repeating this 500,000 times via computer algorithm gave an estimate of the distribution for the proportion of matching abnormalities when the null hypothesis was true.

Statistical analysis was performed using R version 4.0.4 (R Foundation for Statistical Computing, Vienna, Austria) and the Python packages of Scikit-learn [84] and Matplotlib [85].

## Results

**Fig. 4:**
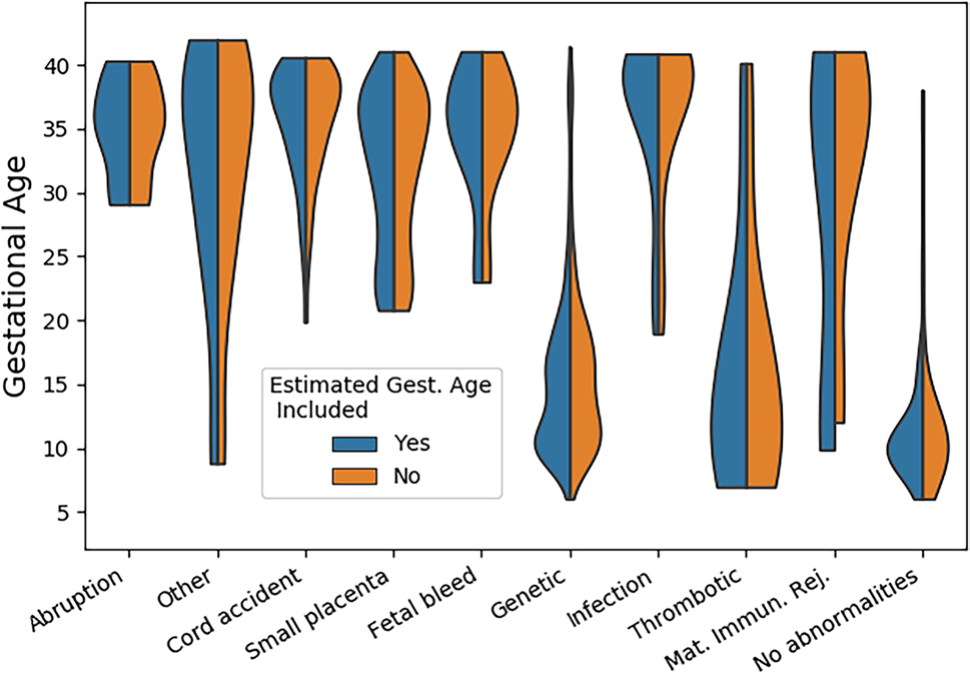
Violin plot of pregnancy pathologies with and without estimated gestational age (GA) cases. The frequencies of the different pathologies remained similar whether cases with estimated GA were included or not in the GA distributions

Of the original 1527 cases, 12 cases were excluded due to an absence of any placental pathology slides (Fig. 1). Two hundred twenty-one cases could not be classified due to absence (*n* = 99) or lack of (*n* = 122) chorionic villi in the placental sample. We estimated the gestational ages of 178 losses. Including these 178 cases did not lead to any visually identifiable change in the violin plot distributions of gestational age for any category of pregnancy loss (Fig. 4). Thirty-eight cases were excluded due to an inability to date the GA at loss by any means. The demographics of the final case series are presented in Table 1.

**Table 1:** Case series demograp﻿hics

Characteristic	Count (%)
Maternal age	
< 20 years	10 (0.8%)
20– < 30 years	210 (17%)
30– < 40 years	841 (67%)
> = 40 years	93 (7.4%)
Data missing	102 (8.1%)
Maternal BMI	
Underweight (< 18 kg/m^2^)	11 (0.9%)
Normal (18– < 25 kg/m^2^)	498 (40%)
Overweight (25– < 30 kg/m^2^)	205 (16%)
Obese (> = 30 mg/m^2^)	140 (11%)
Data missing	402 (32%)
Sex of fetus	
Male	235 (19%)
Female	212 (17%)
Data missing	809 (65%)

Of the 1256 cases analyzed from 922 patients, there were 878 (69.9%) miscarriages and 378 (30.1%) antepartum stillbirths. The average maternal age of these cases at delivery was 33.7 ± 4.8 years (range 14.8 to 48.3 years). A total of 102 cases had no maternal age at delivery in the clinical record. Most pregnancy losses occurred in the first trimester (44.8%) as compared to the second (35.0%) and third trimesters (20.2%), as defined by American College of Obstetricians and Gynecologists (ACOG) criteria [86].

**Fig. 5:**
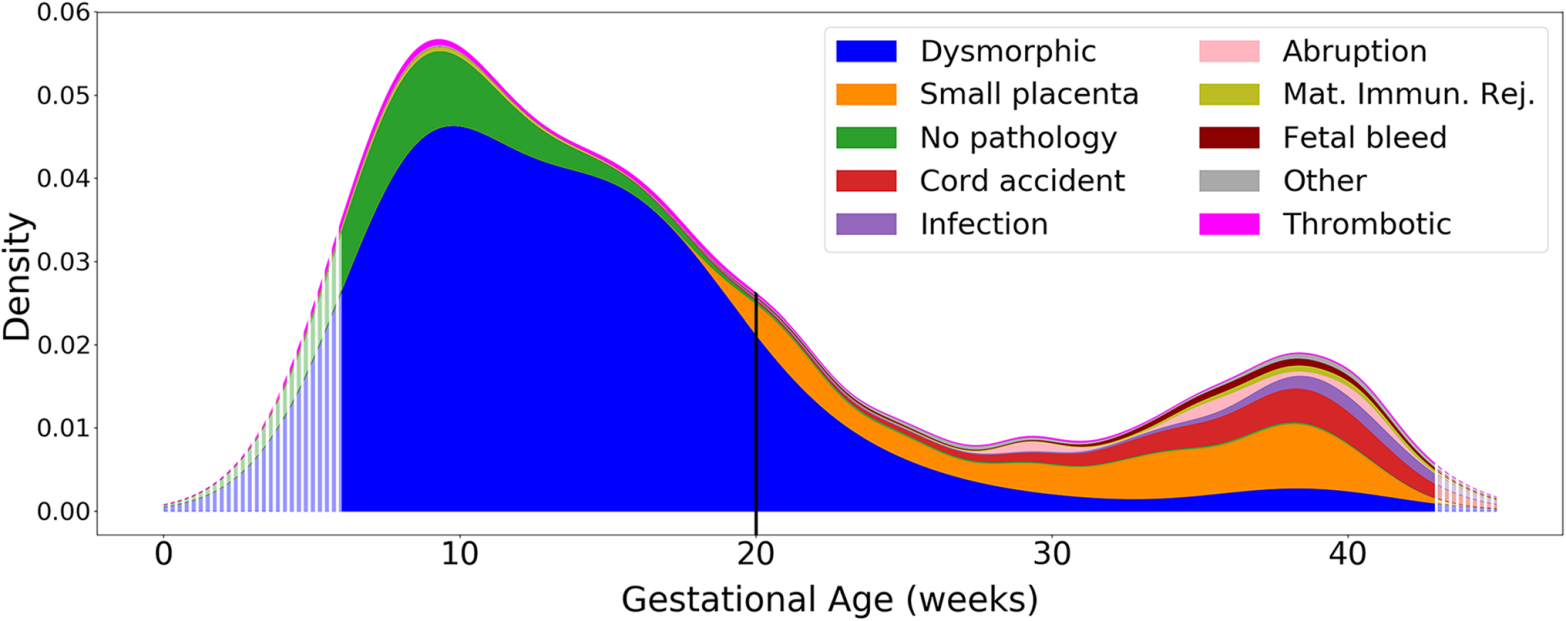
Density plot of pregnancy loss pathologies from 6 to 43 weeks of gestation. Hatched edges represent mathematical extrapolations of the density plot beyond the primary data. The vertical line at 20 weeks represents the demarcation between miscarriages and stillbirths. Mat. Immun. Rej. = maternal immunologic rejection

**Fig. 6:**
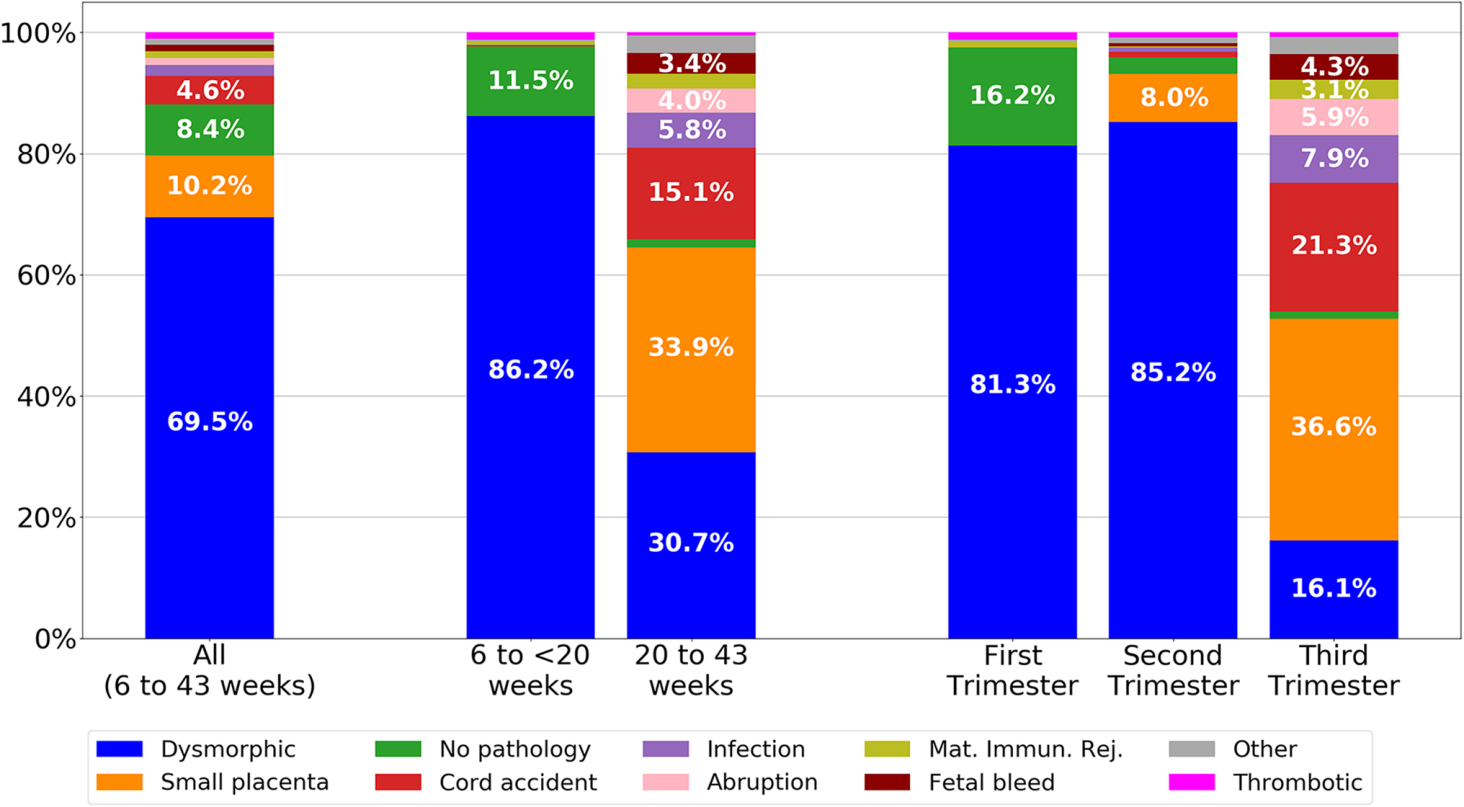
Stacked bar charts of placental pathologies in pregnancy losses. All cases (left bar); miscarriages and stillbirths (middle pair); by trimester (right three bars). Mat. Immun. Rej. = maternal immunologic rejection

The tabulation of percentages of each type of abnormality following the order of our classification system is displayed in Table 2, and a graphical density plot of this data is presented in Fig. 5. Abnormalities were identified in 777/878 (88.5%) of miscarriages (losses prior to 20 weeks of gestation). Seven hundred fifty-seven out of 878 (86.2%) of miscarriages were marked by dysmorphic chorionic villi (Fig. 2), while 111/878 (11.5%) revealed no pathologic findings. In contrast, abnormalities were identified in 373/378 (98.7%) of analyzed stillbirths. The most prevalent abnormalities associated with our 378 cases of antepartum stillbirth were small placenta (128, 33.9%), dysmorphic chorionic villi (116, 30.7%), and cord accidents (57, 15.1%) (Fig. 6). Of the 873 total cases of losses with dysmorphic chorionic villi, 644 (73.8%) cases showed TIs, while 229 (26.2%) showed only trophoblast invaginations.

**Table 2:**
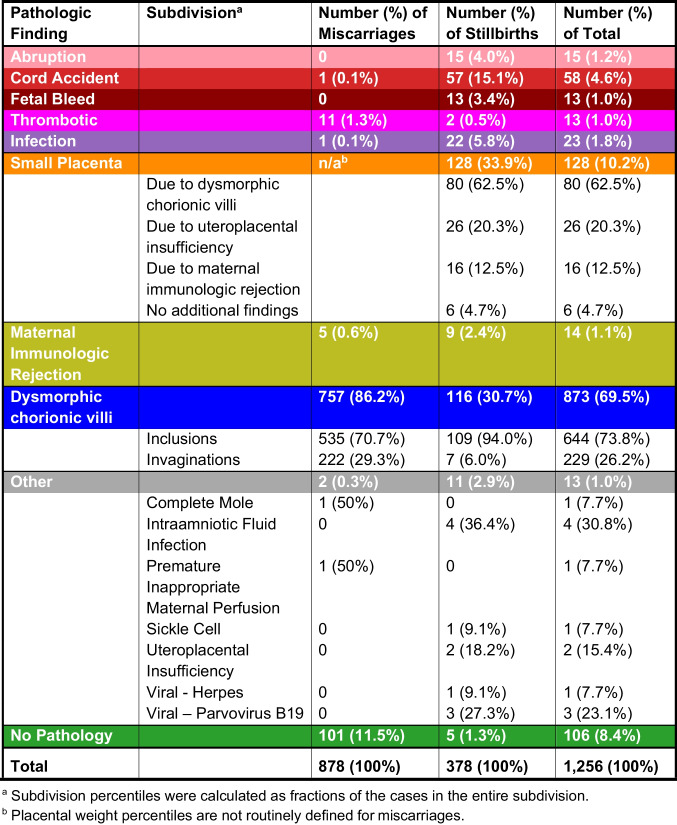
Placental pathologies identified (*n* = 1256)

**Fig. 7:**
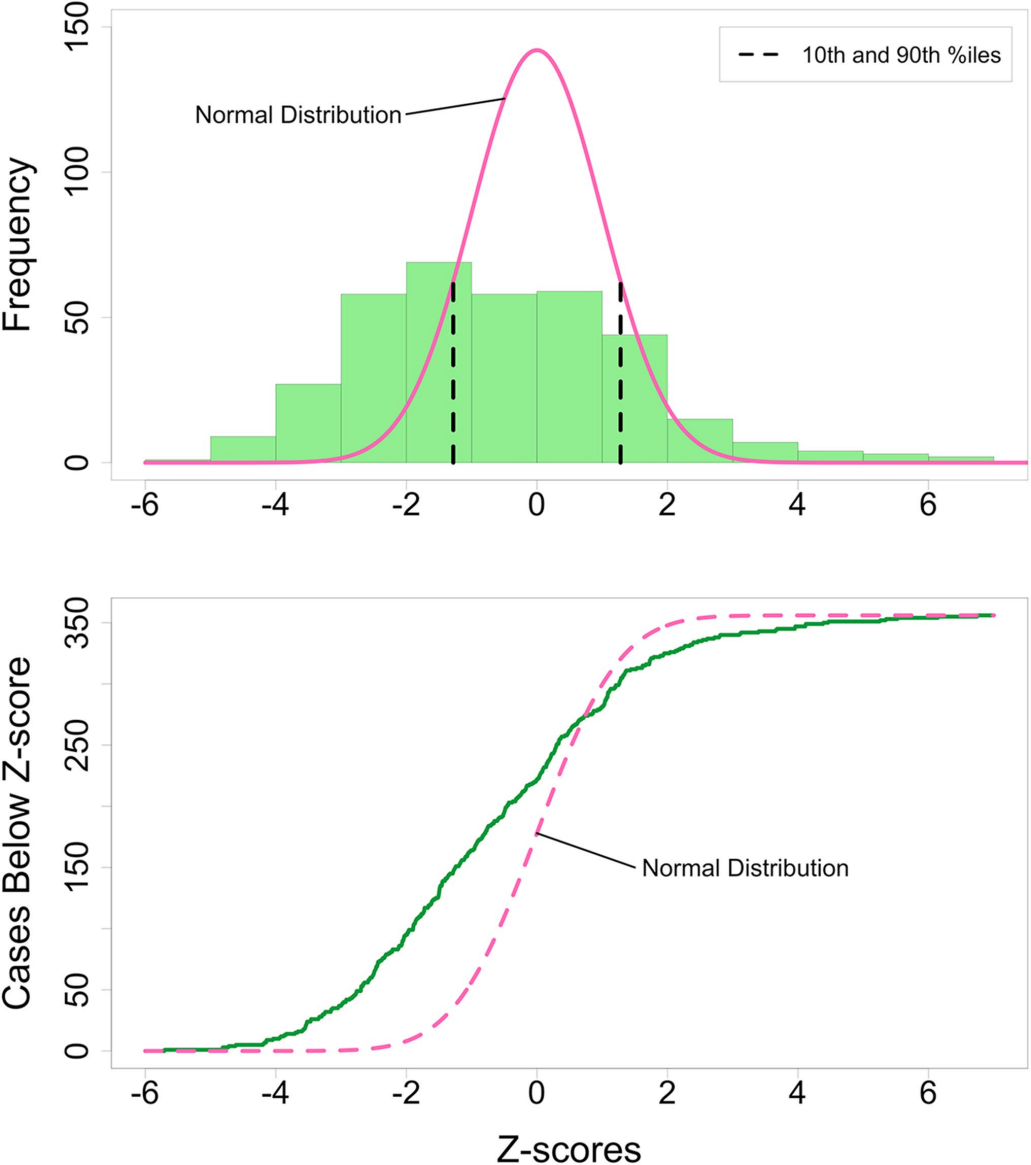
Normal versus loss case series weight distributions. (Upper) Normal placenta *z*-score weight distribution (pink line) compared to this loss case series (green columns). Tenth and 90th percentiles (black dashed lines) are indicated for reference. (Lower) q-q plot to illustrate excess number of small (47 cases) and large (20 cases) placentas (green line) compared to the normal placenta *z*-score weight distribution (pink dashed line). Two large outliers with *z*-scores of + 9 and + 16.6 were not plotted

Placental weights were available in 355/378 (93.9%) of stillbirth cases. Converting percentiles to *z*-scores enabled the visualization of this case series against the standard *z*-score distribution of placentas from uncomplicated term or preterm livebirths [54]. The normal *z*-score distribution of placental weights (pink curve, upper panel Fig. 7) differed from our cases’ stillbirth placental weight distribution (green bars, upper panel Fig. 7), as 47/355 (13.2%) stillbirth cases fell below the normal distribution and 20/355 (5.6%) above (q-q plot, lower panel Fig. 7). The probability that the stillbirths in this case series would demonstrate this degree of dispersion beyond the normal distribution by chance was less than 2 × 10^−16^.

**Fig. 8:**
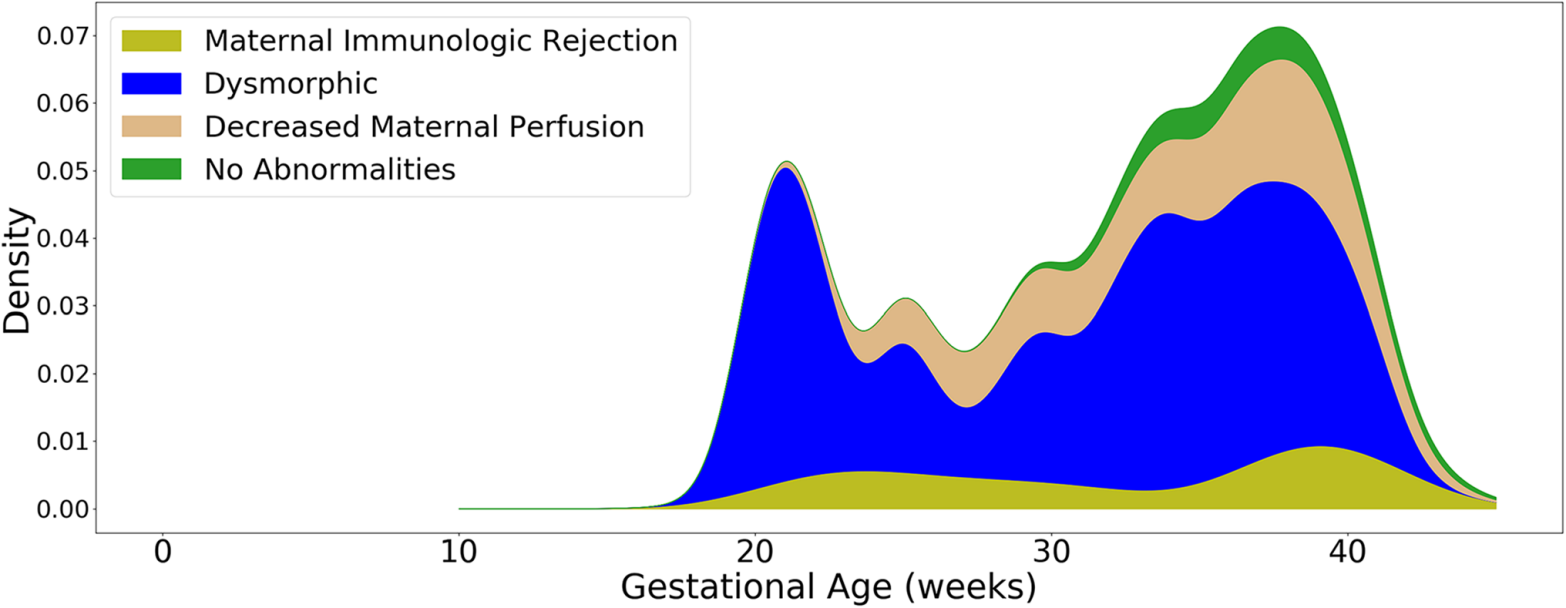
Density plot of small placenta associated pathologies

Within the group of 128 stillbirths with a small placenta, 80 (62.5%) were associated with dysmorphic chorionic villi, 26 (20.3%) with uteroplacental insufficiency, and 16 (12.5%) with maternal immunologic rejection (Fig. 8). Six cases (4.7%) demonstrated no additional pathologic findings. One hundred nine out of 128 (85%) of stillbirths with a small placenta had placental weights that were at or less than the 1st percentile. Within the group of 44 stillbirths with a large placenta (trimmed fixed disk weight greater than the 90th percentile), 20 (45.5%) were associated with dysmorphic chorionic villi, while the remainder were associated other miscellaneous abnormalities.

**Fig. 9:**
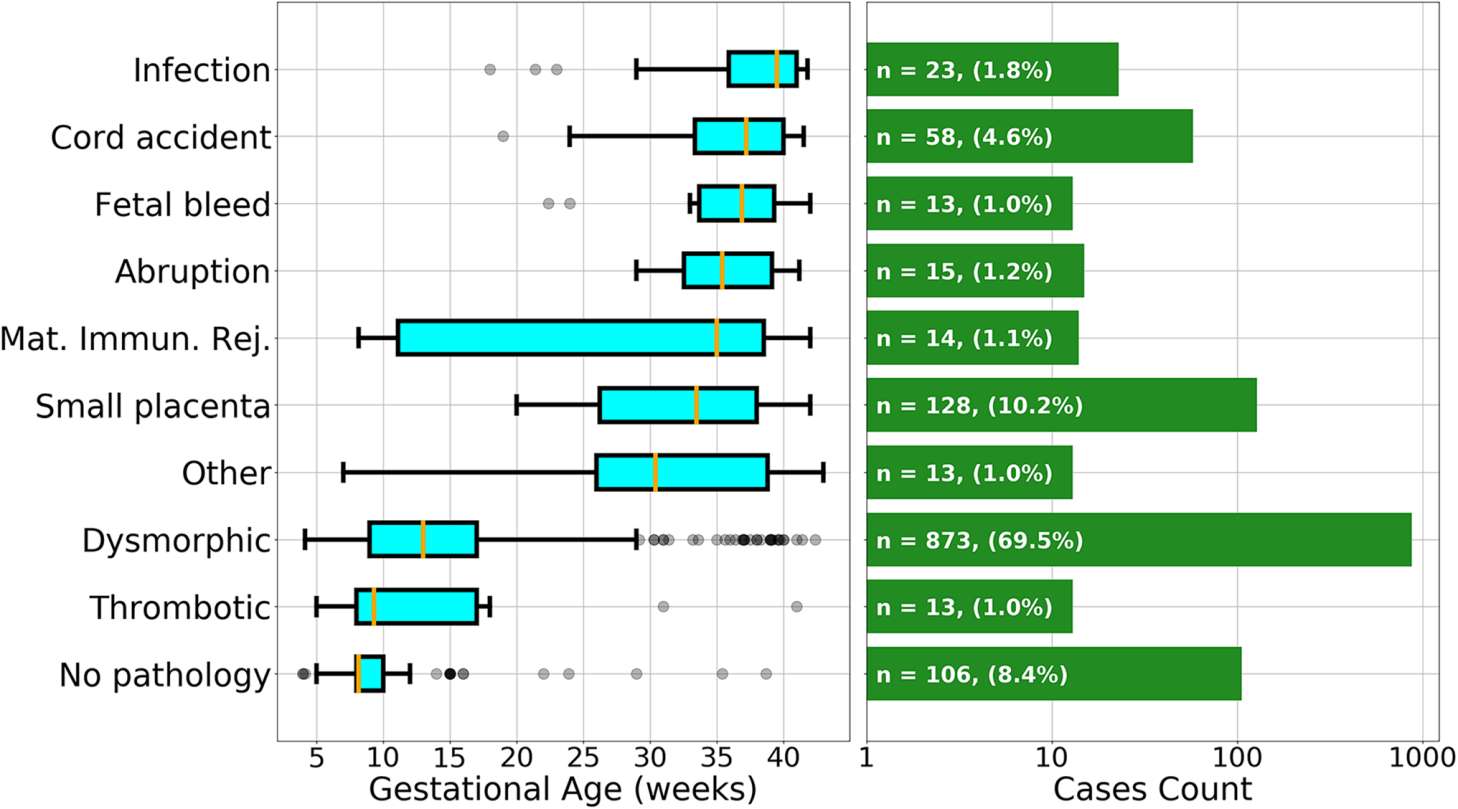
Box plot of pathologic findings in pregnancy loss cases. Gestational age median (gold line), interquartile (25th to 75th percentile) range (teal box), minimum without outliers (lower bar), maximum without outliers (upper bar), and outliers (grey circles) (left panel). Number and percentages of pregnancy loss cases for each pathologic finding (green bars) (right panel). Mat. Immun. Rej. = maternal immunologic rejection

Compared to losses occurring in the first two trimesters, third trimester stillbirths demonstrated increasingly varied abnormalities, with the highest percentage of cases with small placentas (36.2%) and cord accidents (21.2%) (Fig. 6). The median gestational age of loss for each pathologic finding is displayed in Fig. 9. Thrombotic and dysmorphic chorionic villi were the most prevalent associated findings in early pregnancy losses, with medians at 9.3 and 13.0 weeks, respectively. Most other abnormalities were seen later in gestation, such as small placenta at 33.5 weeks, maternal immunologic rejection at 35.0 weeks, abruptions at 35.4 weeks, fetal maternal hemorrhages at 36.9 weeks, cord accidents at 37.2 weeks, and infections at 39.5 weeks.

Two hundred thirty-one out of 922 (25%) patients had more than one loss included in the case series, ranging between 2 (16.8%) and 6 (0.2%) losses. One hundred ninety-one out of 231 patients (82.7%) with more than one loss had two or more losses with the same pathologic findings. The most prevalent recurrent findings were dysmorphic chorionic villi (94.8%), followed by no abnormalities (3.14%).

## Discussion

Utilizing the presented classification system, we identified a pathologic finding in 91.6% of pregnancy losses ranging between 6 and 43 weeks of gestation and 98.7% of stillbirths, underscoring the utility of placental pathologic examination for elucidating potential mechanisms underlying pregnancy loss. As placental pathological examination is already the recommended standard of care following stillbirth [23, 87–92], our expanded methodology may aid clinicians in analyzing previously unexplained or challenging cases.

Our study’s finding that a mechanism for almost 99% of stillbirths could be elucidated by placental examination is a significant improvement compared to other studies. Blythe et al. examined 258 clinically unexplained stillbirths (CUS) using ReCoDe criteria, finding that 60.5% of CUS were due to “placental insufficiency” and/or fetal growth restriction [90]. Specifically, their results showed that ReCoDe category C4 (placenta, “other placental insufficiency”) and C5 (placenta, “other”) were present in 146 (56.5%) cases. Importantly, category C5 included the diagnosis of a small placenta, which was similarly defined as < 10th percentile placental weight for gestational age. Man et al. analyzed the placental pathology of 931 intrauterine fetal demises from 13 to 40 weeks of gestational age and found that 32% of stillbirths were due to abnormalities of the placenta [87]. Another study from the Stillbirth Collaborative Research Network determined that 12.7% of all stillbirths were due to “placental insufficiency” and were, therefore, potentially preventable [93]. A study using TULIP criteria deemed that 27% of stillbirths fell in their placenta cause category [94].

The 20-week marker in the density plot of placental pathologies (Fig. 5) reveals the often-identified U-shaped curve for stillbirth rates [95–97]. Viewing these losses as a continuum, rather than starting at 20 weeks, suggests a more nuanced and improved understanding of the epidemiology of pregnancy losses.

Our finding that a third of previously unexplained stillbirths were associated with a small placenta may be of clinical utility, as prenatal identification of a small placenta may reveal important growth discordance between the fetus and its primary supporting organ [98, 99]. While the Amsterdam criteria defines a placenta with a weight less than the 10th percentile as “placental hypoplasia due to maternal malperfusion,” [45] our data suggest that a placenta can be small for this and other reasons. While placental size alone may not predict stillbirth, we observed an increased number of small placentas in our case series. These results support Hutcheon et al.’s findings that the probability of stillbirth increased significantly with a placental weight more than one standard deviation below the mean [41]. Furthermore, our data contained a significant proportion of extremely small placentas weighing less than the 1st percentile for their gestational age.

Placental size evaluation could provide clinicians with additional data and tools to identify high-risk pregnancies and help determine when to deliver [98–100]. Our case series demonstrated a peak of losses at full term, in line with other studies that demonstrate the prevalence of full-term stillbirths [90, 101]. Although not currently clinically validated, the identification of a fetus with a small placenta, when balanced with other clinical risk factors, may support an earlier delivery to potentially prevent antenatal stillbirth.

TIs and invaginations have been shown to be associated with abnormal genetics, including cases of triploidy, trisomies, and other genetic conditions [27–30, 47, 49]. Therefore, the identification of TIs in most miscarriages suggests a genetic mechanism for these losses [20, 25]. Support for the strong association of developmental anomalies [102] and genetic abnormalities as the basis of pregnancy loss also comes from detailed genetic studies of loss cases [103–110]. However, validation of the specific genetic bases of TIs awaits further, more detailed, genetic analysis of these loss cases.

An increased frequency of TIs and invaginations have been observed in cases of placenta accreta, increta, and percreta [18] and intrauterine growth restriction [20], but not in cases of gestational diabetes, gestational hypertension, or preeclampsia [51]. There is no data relating TI frequencies to other common obstetrical pathologies, such as placenta previa, or to the method of conception. Investigating the relationship between TIs and method of conception, such as in vitro fertilization (IVF) or intracytoplasmic sperm injection (ICSI), would be worthy of future studies.

Our study does not directly address mechanisms underlying the association of TIs with either miscarriages or small placentas, but previous studies on the relationship between cytotrophoblasts and syncytiotrophoblasts may shed some light on this issue [26, 111]. First, syncytiotrophoblasts are created by the fusion of cytotrophoblasts [111]. Second, alterations in the rates of cytotrophoblast proliferation and fusion into the syncytial layer determines the bending of the trophoblast bilayer [26], and increased cytotrophoblast proliferation or decreased fusion leads to inward bending (invagination) of the trophoblast bilayer. Cross sections of chorionic villi through these invaginations create TIs. It should be noted that TIs are epithelial islands within a chorionic villus cross section, not a body within the syncytiotrophoblast cytoplasm (see Fig. 2). Therefore, genetic abnormalities that lead to increased cell proliferation or decreased cell differentiation may lead to increased trophoblast invaginations and inclusions. Although this alteration in the placenta alone may not be deleterious to placental function, other organs in the embryo and fetus may be very susceptible to alterations in branching morphogenesis and infolding, such as the heart [110, 112, 113]. Therefore, further molecular and genetic understanding of the formation of trophoblast invaginations and inclusions may elucidate specific mutations that lead to pregnancy loss.

Although a priori we did not define a separate causal category for stillbirth with a large placenta, we also observed that there was an increased number of large placentas in our case series, indicating a potentially unexplored research avenue.

Our paper’s strengths included the large number of cases examined spanning over a wide gestational age range, as well as the utilization of a classification system for losses that may be elusive to prior classification measures. Although our study was limited by selection bias from the nature of our specialized consultation service, our findings aligned with US national pregnancy loss distributions across the course of pregnancy [6, 16]. In addition, the large number of cases with a small placenta suggests the potential benefit of further research examining the utility of estimated placental volume measurements during clinical care [41, 98, 99].

Our study’s greatest weaknesses were that the sample population was a non-random series of consultative cases, the data was analyzed by a single pathologist at one institution, and gross pathology descriptions relied on materials supplied by referring pathologists. In addition, the gestational age for 178 out of 1256 total cases (14.2%) was approximated. However, this approximation did not appear to significantly affect the results (see Fig. 4). Another limitation was the lack of a comparison group of placentas from livebirths, although pathologic findings in normal placentas have been well studied [114, 115]. We also lacked robust data on maternal demographic and clinical characteristics. For instance, we did not have data on maternal race or ethnicity, which significantly limited us from analyzing this important mediating factor, as, for example, non-Hispanic Black patients have consistently higher rates of fetal demise [16, 116]. While lack of time of death data might also have led to placental weight changes after stillbirth, placental weights for intrapartum versus antepartum stillbirths have not been shown to vary significantly [41]. Lastly, assigning a single abnormality has potential limitations. Incidental findings surely play a contributing and compounding role to the mechanism of any given loss.

## Conclusions

Prior research estimates that up to one-fourth of stillbirths are potentially preventable, most of whose etiology originates in the placenta [88, 93, 117]. Hutcheon et al. concluded their seminal 2012 paper with a clarion call that placental volume measurement may “improve the prenatal identification of fetuses at increased risk of developing adverse perinatal outcomes.” [41]. Our research reinforces this insight with the finding that one-third of previously unexplained stillbirths were associated with a small placenta. We also suggest that these small placentas could have been detected in utero and flagged as high risk prior to the loss. Additionally, we highlight that the identification of dysmorphic chorionic villi containing trophoblast inclusions may be one way to potentially identify genetic abnormalities for further exploration. Adding these two diagnostic categories appears to have eliminated most remaining unexplained loss cases, supporting their adoption and inclusion in pregnancy loss evaluations.

## Data Availability

The data underlying the results presented in the study are available from the Dryad Digital Repository database (https://doi.org/10.5061/dryad.3xsj3txks).
